# Towards a zero-shot low-latency navigation for open surgery augmented reality applications

**DOI:** 10.1007/s11548-025-03480-4

**Published:** 2025-08-05

**Authors:** Michael Schwimmbeck, Serouj Khajarian, Christopher Auer, Thomas Wittenberg, Stefanie Remmele

**Affiliations:** 1 Research Group Medical Technologies, University of Applied Sciences Landshut, Landshut, Germany; 2 Chair for Visual Computing, Friedrich-Alexander-Universität Erlangen-Nürnberg, Erlangen, Germany; 3 Intelligent Embedded Systems Lab, University of Freiburg, Freiburg im Breisgau, Germany; 4 Fraunhofer Institute for Integrated Circuits IIS, Erlangen, Germany

**Keywords:** Augmented reality, HoloLens, Surgical navigation, Segment anything model, Forecasting

## Abstract

**Purpose:**

Augmented reality (AR) enhances surgical navigation by superimposing visible anatomical structures with three-dimensional virtual models using head-mounted displays (HMDs). In particular, interventions such as open liver surgery can benefit from AR navigation, as it aids in identifying and distinguishing tumors and risk structures. However, there is a lack of automatic and markerless methods that are robust against real-world challenges, such as partial occlusion and organ motion.

**Methods:**

We introduce a novel multi-device approach for automatic live navigation in open liver surgery that enhances the visualization and interaction capabilities of a HoloLens 2 HMD through precise and reliable registration using an Intel RealSense RGB-D camera. The intraoperative RGB-D segmentation and the preoperative CT data are utilized to register a virtual liver model to the target anatomy. An AR-prompted Segment Anything Model (SAM) enables robust segmentation of the liver in situ without the need for additional training data. To mitigate algorithmic latency, Double Exponential Smoothing (DES) is applied to forecast registration results.

**Results:**

We conducted a phantom study for open liver surgery, investigating various scenarios of liver motion, viewpoints, and occlusion. The mean registration errors (8.31 mm–18.78 mm TRE) are comparable to those reported in prior work, while our approach demonstrates high success rates even for high occlusion factors and strong motion. Using forecasting, we bypassed the algorithmic latency of 79.8 ms per frame, with median forecasting errors below 2 mms and 1.5 degrees between the quaternions.

**Conclusion:**

To our knowledge, this is the first work to approach markerless in situ visualization by combining a multi-device method with forecasting and a foundation model for segmentation and tracking. This enables a more reliable and precise AR registration of surgical targets with low latency. Our approach can be applied to other surgical applications and AR hardware with minimal effort.

## Introduction

Applying augmented reality (AR) navigation to surgical applications offers decisive benefits. In specific, optical see-through head-mounted displays (OST-HMDs), such as the Microsoft HoloLens 2, have been emerging in surgical navigation tasks [1]. Using AR, a preoperatively obtained Computer Tomography (CT) model of the target anatomy can be projected as a three-dimensional virtual model onto its in situ counterpart. This provides ergonomic benefits to surgeons, who no longer need to shift their focus between the intraoperative situs and the planning data during interventional procedures. Furthermore, seeing the surgical situs superimposed with spatial organ relationships and internal organ structures can enhance the accuracy and speed of the surgical intervention [1, 2].

Despite the progress and availability of minimally invasive surgical methods, open liver surgery remains highly relevant, specifically for complex tumor resections [3]. However, distinguishing tumors and vessels poses additional challenges, increasing the risk of non-optimal resections. Sonography is mainly utilized for intraoperative orientation and navigation in today’s clinical practice, but it requires substantial expertise for image interpretation and spatial reconstruction [4]. Hence, open liver interventions could greatly benefit from navigation technologies such as AR [2].

Some initial studies already investigated AR navigation for open liver surgery [1, 4–6]; however, there remains a lack of fully automated and markerless approaches that would alleviate the integration into clinical workflows. Two state-of-the-art works closest to solving these challenges are those of Golse et al. [5] and Khajarian et al. [6].

Golse et al. [5] introduced a markerless navigation method based on a single Intel RealSense RGB-D camera. The system’s setup and preparation time was under 10 min, and the registration accuracy reached as low as 7.9 mm root-mean-squared error, as evaluated in a clinical study. However, major limitations of the approach are the 2D-only visualization on a flat screen as well as distortions in organ segmentation and global pre-registration under conditions of severe occlusions.

Khajarian et al. [6] proposed an automatic method utilizing the HoloLens 2 sensor data. The rigid registration of a pre-operative liver model onto the respective target structures is based on point clouds cropped from depth maps. However, prior work has highlighted limitations in the accuracy of the HoloLens 2 depth sensor, which restricts the device’s applicability in high-precision medical applications [7]. Furthermore, the approach employs a specialized machine-learning-based segmentation model for live organ tracking, which must be trained for each specific application, each requiring a significant amount of annotated training data. In domains such as open liver surgery, it is difficult to obtain these data in sufficient quantity and quality. If the lack of adequate training data results in poor segmentation quality, this consequently leads to inaccurate (rigid) registration. This, in turn, hampers the subsequent non-rigid registration to the deformable organ, which strongly depends on the quality of the preceding rigid alignment [8].

To overcome the limited applicability and high preparation effort required to prepare specialized segmentation models for each application, the foundation model *Segment Anything Model* (SAM) [9] offers zero-shot performance across a wide range of tasks and supports application-specific constraints through various prompts. Recent studies applying SAM to clinical open liver surgery data demonstrated promising segmentation results [10, 11]. However, visual prompt engineering—such as placing ’positive’ and ’negative’ seed points in the scene combined with iterative re-prompting—remains essential for achieving stable performance in clinical applications.

In summary, an optimal solution for AR-based navigation in open liver surgery depends on (1) live organ tracking and segmentation and (2) robust registration of the pre-operative organ model to the intraoperative situs. Hence, the system must capture the organ structure as accurately as possible for robust rigid alignment and subsequent non-rigid refinement (not part of this work), even under occlusions, while remaining broadly applicable in a scarce-data domain. The presented methodology addresses these requirements and further tackles the key challenge of latency caused by high inference times in the prior work. Our contributions are summarized as follows:

1. We introduce a proof-of-concept for a novel multi-device approach for automatic and markerless AR navigation in open liver surgery. Our method involves a preparation effort of only a few seconds and combines the visualization and interaction capabilities of a HoloLens 2 HMD with a precise and reliable registration using an Intel RealSense RGB-D camera. By leveraging the SAM foundation model as the segmentation component—prompted in a user-controlled manner directly within the AR environment—the entire processing pipeline is transferable to other surgical applications without requiring additional training data.
2. We investigate Double Exponential Smoothing as a forecasting technique for registration transforms to reduce latency caused by the high inference times of our method and comparable works. Thus, we mitigate delays in the visualization for the user.
3. We evaluate the registration quality of the proposed method through a liver phantom study that simulates real-world challenges such as occlusion, motion, and viewpoint variation. The phantom study demonstrates the practical feasibility of our approach, which is required prior to clinical studies.

**Fig. 1:**
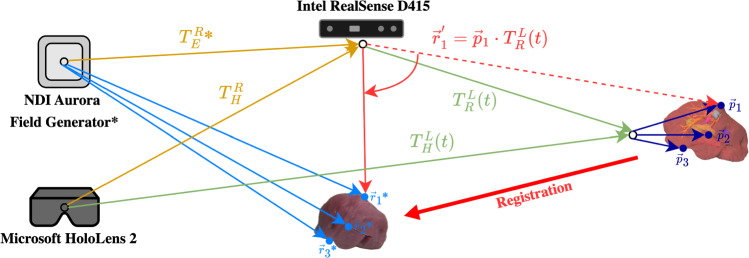
Our proposed navigation method is based on the Intel RealSense RGB-D camera (R) and visualizes the registered virtual model (L) on a Microsoft HoloLens 2 HMD (H). This setup provides a 3D view persistent through the user’s environment. The RealSense-HoloLens perspectives were initially calibrated using an ArUco chart, and the resulting transformation $T_{H}^{R}$ is iteratively refined using the HoloLens inside-out tracking. To evaluate registration accuracy, we defined three reference points $\vec {p}_{i}$ on the virtual liver model. Corresponding points $\vec {r}_{i}$ were measured on the physical liver phantom using an NDI Aurora electromagnetic tracking system (EMS, E). The RealSense-EMS perspectives were calibrated using the corner points of an ArUco chart. Rationale of the included transforms are described in Sections Phase 1: preparation, Phase 2: tracking and registration, and Registration accuracy with respect to occlusion. Components marked with an asterisk (*) are required only for evaluation

## Methods

**Fig. 2:**
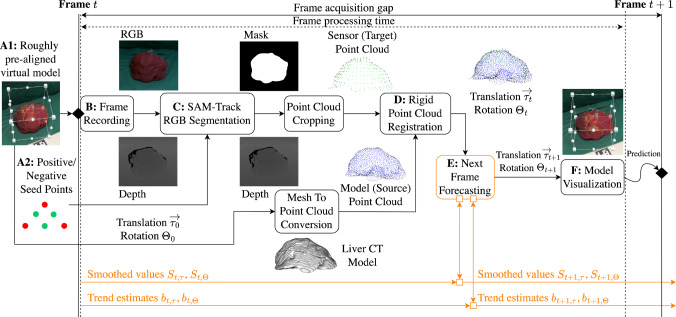
Overview denoting successive process steps. RGB and depth data are acquired (B) using an Intel RealSense camera. Live segmentation (C) of the target organ, based on the SAM-Track approach, is initialized by the manual choice of six seed points in AR (A2). A sensor point cloud (target) is cropped from the total point cloud using the resulting segmentation mask, and a model point cloud is derived from the organ’s CT scan (source). Following an initial pre-alignment (A1) performed by the user, the source point cloud is registered onto the target point cloud (D) in all consecutive frames. Based on the resulting transformation, algorithmic latency is bypassed by forecasting (E, orange) one frame ahead. A virtual 3D model of the CT volume is projected (F) at the registered position using Microsoft HoloLens 2

As indicated in Fig. 1, our approach decouples the visualization from the registration, as AR HMDs currently have limitations in terms of precise depth sensing. Hence, we utilize the HoloLens 2 HMD (Microsoft) for visualization and interaction, while employing the RealSense D415 RGB-D camera (Intel) as a high-definition 3D-sensing device. Figure 2 consolidates our process to establish a foundation model based and low-latency AR navigation method applied to open liver surgery. The individual phases and steps are described in the following subsections.

### Phase 1: preparation

To enable robust segmentation and registration even in challenging clinical situations, we propose an approach with minimal user interaction comprising two steps, which require approximately 20 s in total.

(1) At present, automatic global registration of the liver organ is still challenging due to the lack of distinct features, especially under high levels of occlusion [5, 6]. However, this registration is crucial for later clinical practice and creates a significant demand for quality control. Hence, the surgeon is instructed to roughly pre-position a virtual model of the liver’s CT scan to its real-world counterpart (Fig. 2, *step A1*), ensuring a maximum distance of approximately 10 cm between the visible margins. We provide the intuitive HoloLens 2 hand interface to enable a rapid pre-alignment between both structures. The subsequent initial transform ($T_{H}^{L}(0)$) of the virtual liver model (L) substitutes for a global registration from the perspective of the HoloLens (H), while incorporating the surgeon’s expertise regarding the organ’s structure.

(2) Establishing robust segmentation of organs is challenging, especially in complex clinical scenarios involving a liver with an irregular or split structure. To better guide the unchanged SAM foundation model in assisting with this task, prior information in the form of prompts is required. Hence, we instruct the surgeon to place six seed points $s_{i}$ directly in AR, each three ones onto the target organ (’positive’), and onto its surroundings (’negative’) (Fig. 2, *step A2*). Similar to [12], we developed a HoloLens 2 application enabling the user to select arbitrary seed points using finger tracking.

### Phase 2: tracking and registration

We acquire high-resolution RGB and depth streams from the Intel RealSense and stream these data to a workstation (Fig. 2, *step B*). In the initial frame, the offset transform $T_{H}^{R}$ between the HoloLens (H) and the RealSense (R) is calibrated using an ArUco chart [13] (C) placed in the field of view of both sensors. Hence, $T_{H}^{R}$ is calculated as $(T_{R}^{C})^{-1} \cdot T_{H}^{C}$. The ArUco chart can be removed after recording the first frame due to the inside-out tracking capabilities of the HoloLens.

To detect the liver in each RGB frame, we employ the SAM-Track approach (unpublished work by Cheng et al.) (Fig. 2, *step C*), which builds upon SAM [9] by incorporating the *Decoupling Features in Hierarchical Propagation* (DeAOT) tracker [14] to achieve high frame rates. We utilize the RGB image coordinates of the three ’positive’ and the three ’negative’ seed points $s_{i}$, placed in *step A2*, as SAM prompts. Each consecutive RGB frame receives a segmentation mask by tracking the initial SAM segmentation using the DeAOT tracker. For performance purposes, our method comprises SAM with a ViT-B backbone and DeAOT with a DeAOTT backbone. Using the depth map from the RealSense camera, a target point cloud is cropped from the total point cloud based on the obtained segmentation mask.

The source point cloud from the liver’s CT model is constructed by sampling 5,000 points and aligned with the target point cloud through rigid point cloud registration (Fig. 2, *step D*). Similar to [6], we employed a voxel resolution of 5 mm for point clouds, balancing structural and temporal resolution. The registration is initialized with the manual pre-positioning (Fig. 2, *step A1*), described by $T_{R}^{L}(0) = T_{H}^{L}(0) \cdot (T_{H}^{R})^{-1}$. In all consecutive frames, the PointToPoint ICP algorithm [15] iteratively updates $T_{R}^{L}(t)$ using a distance threshold of 1 cm.

We visualize the registration results on the HoloLens (Fig. 2, *step F*) by transmitting the registration transformation matrix $T_{R}^{L}(t)$ to the HoloLens app via wireless local network. The app displays a semi-transparent version of the CT model used for registration, which is transformed by $T_{H}^{L}(t) = T_{R}^{L}(t) \cdot T_{H}^{R}$.

### Phase 3: forecasting

Our proposed data processing pipeline operates with a total latency of approximately 100 ms on the available hardware. As in situ navigation is time critical, the related latency must be as low as possible. Due to its importance in time-series analysis and prior conceptual work demonstrating promising results [16], we investigate *Double Exponential Smoothing* (DES) [17] (chapter 6.4.3.3) to forecast the next frame’s transformation (Fig. 2, *step E)*. The process result is thereby pre-computed for the consecutive frame, and algorithmic latency is bypassed as a consequence. We translate DES to registration transforms as follows:

1 $$\begin{aligned}  &   \begin{aligned} S_{{t,\tau }} =&\,\alpha _{\tau } \cdot \vec {\tau }_{t} + (1 - \alpha _{\tau } ) \cdot (S_{{t - 1,\tau }} + b_{{t - 1,\tau }} ) \\ \end{aligned} \end{aligned}$$

2 $$\begin{aligned}  &   \begin{aligned} b_{{t,\tau }} =&\,\gamma _{\tau } \cdot (S_{{t,\tau }} - S_{{t - 1,\tau }} ) + (1 - \gamma _{\tau } ) \cdot b_{{t - 1,\tau }} \\ \end{aligned} \end{aligned}$$

3 $$\begin{aligned}  &   \begin{aligned} S_{{t,\Theta }} =&\,\alpha _{\Theta } \cdot \Theta _{t} + (1 - \alpha _{\Theta } ) \cdot (S_{{t - 1,\Theta }} \cdot b_{{t - 1,\Theta }} ) \\ \end{aligned} \end{aligned}$$

4 $$\begin{aligned}  &   \begin{aligned} b_{{t,\Theta }} =&\,\gamma _{\Theta } \cdot (S_{{t - 1,\Theta }}^{{ - 1}} \cdot S_{{t,\Theta }} ) + (1 - \gamma _{\Theta } ) \cdot b_{{t - 1,\Theta }} \\ \end{aligned} \end{aligned}$$

Forecasting is applied to the translation vector $\vec {\tau }_{t}$ and the rotation quaternion $\Theta _{t}$, both decomposed from a registration transformation matrix *T* at time *t*. For each time step, the smoothed value $S_{t}$ and a trend estimate $b_{t}$ are calculated. We compute $\vec {\tau }_{t+1} = S_{t,\tau } + m \cdot b_{t,\tau }$ using values calculated by Equation (1) and (2), applying linear interpolation. Similarly, we compute $\Theta _{t+1} = S_{t,\Theta } \cdot {b_{t,\Theta }}^{m}$ using values obtained from Equation (3) and (4), applying spherical linear interpolation to rotation quaternions. For $m = 1$ time step, we predict the next frame after the frame acquisition gap interval. Forecasting begins with the second frame, where initial values are set as $S_{0,\tau } = \vec {\tau }_{0}$ and $b_{0,\tau } = \vec {\tau }_{1} - \vec {\tau }_{0}$, as well as $S_{0,\Theta } = \Theta _{0}$ and $b_{0,\Theta } = \Theta _{0}^{-1} \cdot \Theta _{1}$.

The data smoothing factor $\alpha$ and trend smoothing factor $\gamma$ are optimized via a design-of-experiments study that minimizes the deviation between the forecasted and ground-truth transformation matrices. This deviation is quantified in terms of a Translation Error (TE in Euclidean Distance) and a Rotation Error (RE in Angular Distance). We determine TE and RE for each combination of $\alpha$ and $\gamma$ (ranging from 0.0 to 1.0 in increments of 0.1) across three motion scenarios involving a mechanical turning wheel with rotation velocities of 30/15/10 s per turn, respectively. By the high rotation speeds, we simulate rapid organ or sensor movements that may occur during surgical interventions. The liver phantom is positioned on the turning wheel, with its coordinate system off-center, such that the phantom undergoes both rotations and translations. As a reference, we attach an NDI Aurora 5DoF catheter that is tracked using the NDI Aurora system to measure a ground-truth transformation, sampled in accordance with the total latency of our navigation method (100 ms). To optimize the observations to the given problem, we perform regression analysis to estimate the two underlying model functions describing TE and RE dependent on the influencing factors $\alpha$ and $\gamma$. We follow the response surface method outlined in [17] (chapter 5.3.3.6—quadratic model, cubic terms neglected). Minimizing these model functions yields the optimum values for $\alpha$ and $\gamma$ w.r.t. the real-world reference, as provided in Table 1. These optimized hyperparameters are then substituted into Equation (1)–Equation (4).

**Table 1:** Response surfaces for the forecasting translation ($\tau$) and rotation ($\Theta$) errors in response to the Double Exponential Smoothing hyperparameters $\alpha$ (data smoothing factor) and $\gamma$ (trend smoothing factor). The errors represent the difference between the forecasted and ground-truth registration results, based on reference measurements from an electromagnetic tracking system. The optimum hyperparameter values result from the minima of the response surfaces (indicated by points)

Motion Scenario	Translation Error /Euclidean Distance (mm)	Rotation Error /Angular Distance ($^{\circ }$ between quaternions)	Optimum Hyperparameters
30 s/turn			$\alpha _{\tau }=0.7$ $\gamma _{\tau }=0.7$ $\alpha _{\Theta }=0.7$ $\gamma _{\Theta }=0.3$
15 s/turn			$\alpha _{\tau }=0.7$ $\gamma _{\tau }=0.7$ $\alpha _{\Theta }=0.7$ $\gamma _{\Theta }=0.5$
10 s/turn			$\alpha _{\tau }=0.7$ $\gamma _{\tau }=0.7$ $\alpha _{\Theta }=0.8$ $\gamma _{\Theta }=0.6$

## Experiments

We built an experimental setup replicating a scenario for open liver surgery, which includes a rigid silicone phantom of a liver (sampled from [18]) as well as its segmented CT scan. To be comparable to the work of Golse et al. [5], we positioned the liver phantom’s surface approximately 40 cm away from the RealSense camera in each experiment. For data and image processing, we utilized a workstation equipped with a 12th Gen Intel Core i7 CPU and an NVIDIA GeForce RTX 3090 GPU.

Based on this experimental setup, we evaluated diverse real-world scenarios, as described in the following subsections.

### Registration accuracy with respect to occlusion

For the validation of registration accuracy, we determined the Target Registration Error (TRE) using three ($N_p = 3$) reference points on the surface of the liver. In Fig. 1, these points are denoted as $\vec {p}_{i}$ relative to the RealSense camera and $\vec {r}_{i}$ relative to the electromagnetic tracking system (EMS, NDI Aurora), which was used for reference measurements in our experiments. The RealSense-EMS perspectives were aligned ($T_{E}^{R}$) using the fiducial registration method of Horn [19], based on the image-determined corners of an ArUco chart. From the EMS perspective, both the corners of the ArUco chart and the landmarks on the phantom $\vec {r}_{i}$ were located using an NDI Aurora 5DoF catheter. Ultimately, the TRE of the registration transform $T_{R}^{L}(t)$ was calculated using Equation (5), with the error expressed as Euclidean Distance (ED):

5 $$\begin{aligned} \text {TRE}(t) = \sqrt{\left( \frac{1}{N_p}\right) \cdot \sum _{i=1}^{N_p} \text {ED}\left( \vec {r}_{i}, \vec {p}_{i} \cdot T_{R}^{L}(t) \cdot T_{E}^{R} \right) ^{2}} \end{aligned}$$

Prior work [5, 6] motivated the development of navigation methods capable of handling significant occlusions, which are common in clinical scenarios. Therefore, we tested our proposed method across six different occlusion cases that simulate established open liver surgery resection scenarios [6]. A test user (M.S.), experienced with the HoloLens interaction methods, conducted ten repetitions for each occlusion scenario to investigate the impact of various pre-alignments on the TRE and success rates. Success rates were calculated as described in [6]. However, registration accuracy heavily depends on the quality of the target point cloud, which is cropped using the liver segmentation mask. Hence, we further computed the Intersection-over-Union (IoU) to assess the segmentation accuracy of the SAM model w.r.t. a reference annotation of a human rater. The ratio of visible pixels of the liver phantom in relation to the non-occluded reference scenario provides a measure of occlusion (visibility factor).

### Registration accuracy with respect to motion and viewpoints

To evaluate how different types of motion affect registration quality, we applied our method to four motion scenarios. In each scenario, the liver phantom was rotated again using the mechanical turning wheel at varying velocities (30/15/10/0 s per turn). After every 90-degree turn, the TRE of previously selected reference points (frontal surface) was obtained as described in the previous subsection (i.e., extrinsic data). Dynamically computing the TRE during motion is challenging due to the attached EMS sensors. Hence, the deviation over time between the aligned source and target point clouds was monitored using the Chamfer Distance (i.e., intrinsic data). Thus, this experiment also assesses how accurately points are registered if they are distant from the registered surface, ultimately at the opposite side, thus simulating potential tumor or vessel positions. Chamfer Distances were averaged over 300 frames each. Additionally, computing the success rate [6] investigates the stability of the registration over the entire motion test series.

### Forecasting accuracy with respect to motion and forecasting hyperparameters

Since our method involves forecasting translation and rotation information, we further quantified the forecasting error relative to the registration result of the subsequent frame, averaged over 300 frames each. The four motion scenarios described in the previous subsection were used for this analysis. We compared (1) the real and forecasted translation vector by the Euclidean Distance [mm] and (2) the real and forecasted rotation quaternion by the Angular Distance [$^{\circ }$ between quaternions]. As we have determined different optimum hyperparameter combinations of $\alpha$ and $\gamma$ across the hyperparameter fitting scenarios (Sect.  Phase 3: forecasting), we investigated each hyperparameter combination within each motion scenario.

## Results

Results on registration and segmentation accuracy, dependent on the occlusion scenarios, are presented in Table 2. The averaged TRE values for scenarios with organ visibility greater than 50 % (comparable to the prior work) ranged between 8.31 mm and 13.52 mm and were thus comparable to the root-mean-squared errors reported by Golse et al. (7.50 mm–13.59 mm) [5]. Registration success rates consistently reached 100 % across all scenarios, outperforming the success rates reported by Khajarian et al. [6]. The SAM segmentation accuracy exceeded 97 % IoU in all investigated scenarios, ensuring precise tracking of the target structure.

**Table 2:** Registration accuracy and success rates for different occlusion scenarios common in open liver surgery. We computed the Target Registration Error (TRE) with respect to reference measurements from an electromagnetic tracking system (NDI Aurora) and evaluated the segmentation accuracy in terms of Intersection-over-Union (IoU). The visibility factor represents the ratio of the visible pixels of the liver phantom in relation to the reference perspective

	Reference	ERH^1^	RH^1^	ELH^1^	LH^1^	LL^1^
						
Visibility Factor/%	100	66	35	85	52	15
Registration Accuracy/TRE (mm)	$9.66 \pm$ 0.97	$8.31 \pm$ 2.20	$11.51 \pm$ 1.55	$10.33 \pm$ 1.24	$13.52 \pm$ 2.78	$18.78 \pm$ 2.01
Segmentation Accuracy/IoU	$0.983 \pm$ 0.001	$0.978 \pm$ 0.001	$0.977 \pm$ 0.001	$0.982 \pm$ 0.001	$0.983 \pm$ 0.002	$0.971 \pm$ 0.001
Success rate/% (Khajarian et al. [6])	100	100	95.04	53.5	3.66	0
Success rate/% (Ours)	100	100	100	100	100	100

^1^ERH: Extended right hepatectomy, RH: Right hepatectomy, ELH: Extended left hepatectomy, LH: Left hepatectomy, LL: Left lobectomy

**Table 3:** Registration accuracy in terms of the Chamfer Distance between the detected and registered point clouds. Results are provided for different motion scenarios, averaged over 300 frames each

	0 s/turn	30 s/turn	15 s/turn	10 s/turn
Registration Accuracy/Chamfer Distance (mm)	$3.03\pm 0.22$	$3.32\pm 0.59$	$3.65\pm 0.50$	$3.61\pm 0.55$

**Table 4:** Registration accuracy dependent on different views on the liver phantom. We computed the Target Registration Error (TRE) using three landmark points (LM arrow) located on a specific part of the phantom’s surface

	Frontal view	Right lobe view	Rear view	Left lobe view
				
Registration Accuracy/TRE (mm)	8.59	14.37	16.07	9.35

**Fig. 3:**
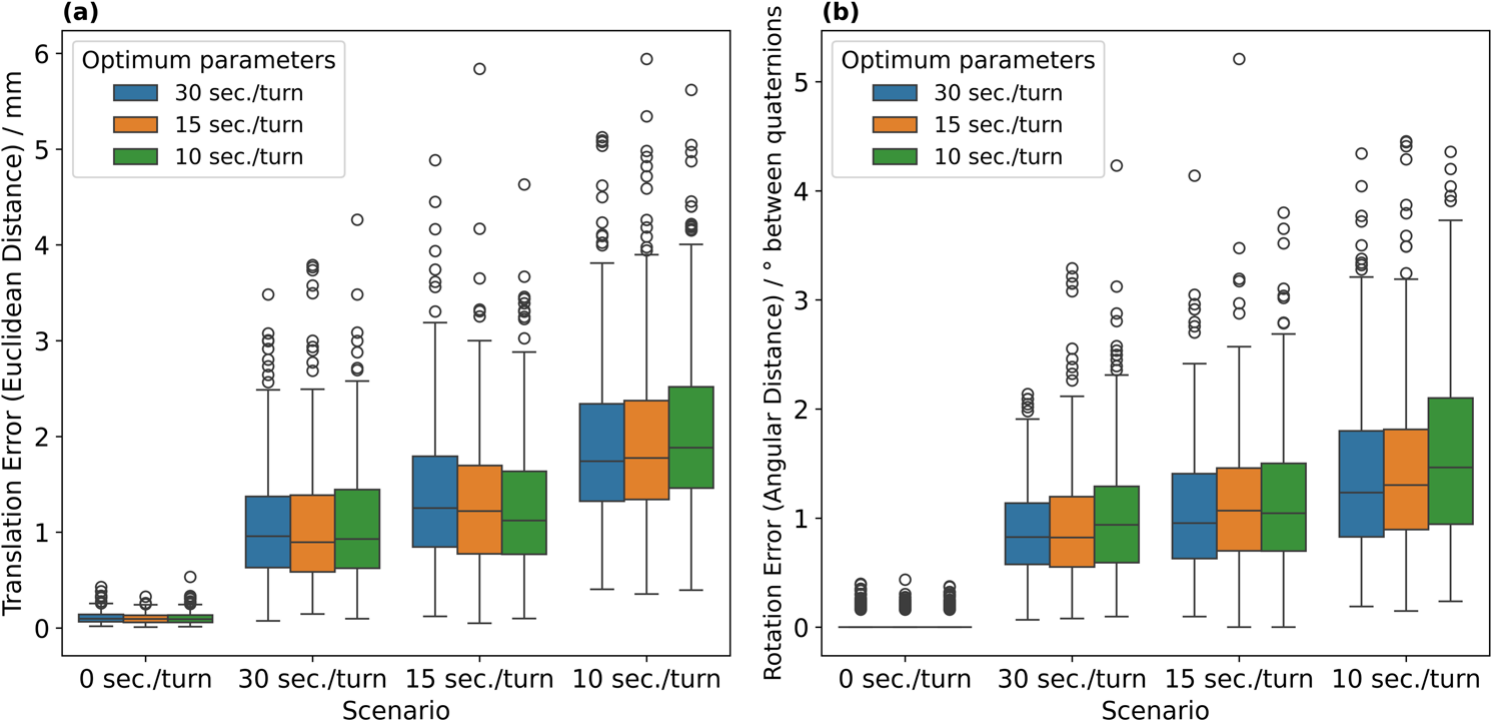
Forecasting translation **a** and rotation **b** errors of our method across four motion scenarios. We applied the optimum forecasting hyperparameters (Table 1) to each scenario, resulting in sub-millimeter and sub-degree deviations across all hyperparameter combinations. Median translation errors were less than 2 mm, and median rotation errors were less than $1.5^{\circ }$ between quaternions in all test series, while algorithmic latency was reduced by 79.8 ms

Mean Chamfer Distances w.r.t. four motion scenarios are presented in Table 3. There were sub-millimeter deviations across all scenarios, while registration remained stable throughout each test series (success rate = 100 %).

Table 4 provides registration accuracy in relation to different viewpoints on the liver phantom and, consequently, the distance to the reference landmark points (LM). Landmark points were marked on a specific part of the phantom’s surface and were barely visible or completely obscured from other perspectives. However, even if the landmark points were located on the opposite side (rear view) of the registered surface, the error increased by only 7.48 mm.

We investigated the forecasting accuracy of our navigation method across different motion scenarios (Fig. 3), dependent on the optimum hyperparameter combinations that were determined using ground-truth measurements (Table 1). Our results demonstrate median translation errors of less than 2 mm and median rotation errors of less than $1.5^{\circ }$ between quaternions in all test series. Additionally, forecasting errors showed only sub-millimeter and sub-degree deviations across the optimum hyperparameter combinations.

By using forecasting, we were able to bypass a computational delay of 4.2 ms for frame recording, 32.1 ms for segmentation and tracking, 43.5 ms for registration, and thus a total of 79.8 ms algorithmic latency.

## Discussion

We investigated registration performance under six different degrees of occlusion and obtained registration errors, which are comparable to the prior work [5]. However, our proposed method offers significant advantages in terms of (1) reliability and robustness, (2) transferability to other surgical applications, and (3) reduced preparation effort. Furthermore, even under high occlusion factors, our method achieved registration success rates of 100 % across all investigated scenarios. This provides decisive benefits during open surgeries for left hepatectomies and lobectomies, which have faced challenges in previous studies [6]. Additionally, the high registration success rates observed during motion scenarios demonstrate the robustness of our method against rapid movements, while the mean Chamfer Distance was consistently below 4 mm.

However, standardized accuracy values from the clinical domain are not yet available. One prior study reported a target accuracy of 3 mm to ensure adequate margins [20]. While the accuracy of our method may be suitable for spatial perception and organ localization, it is insufficient for visualizing blood vessels with millimeter precision. Future work involves the adaptation to non-rigid registration, which is a crucial next step as the liver undergoes substantial deformations during open liver surgery.

Our forecasting approach bypasses 79.8 ms of algorithmic latency in our experiments, which is relevant given the increasing inference times associated with subsequent non-rigid registration. Comparable studies encounter even higher latency (for instance, greater than 200 ms [6]), which increases the demand for forecasting. However, some latency remains due to network transfer delays between the HoloLens and the workstation. Future work needs to focus on addressing such irregular latency issues. Interpolating forecasting results and increasing the forecasting horizon enables to increase the frame rate as a next step.

Furthermore, SAM was combined with the DeAOT tracker in our study to propagate segmentation results to subsequent frames. However, initializing SAM with a representative view on the target organ is crucial to prevent an ambiguous mask that could distort tracker initialization, thereby affecting all downstream segmentation masks. In clinical practice, substantial and abrupt changes in the dynamic surgical scene can occur, for instance temporary full occlusion, coagulation smoke, or surgical tools entering the scene. The robustness of our method against these factors needs to be thoroughly investigated in future work.

In summary, our results demonstrate that the proposed AR-based navigation method offers a more reliable and low-latency solution for open liver surgery and achieves registration performance comparable to state-of-the-art methods. The use of an additional high-resolution depth sensor enables live non-rigid refinement of the registration as a next step. Finally, due to SAM prompt engineering, it can be assumed that our method is adaptable to other surgical applications by simply exchanging the pre-operative organ model.
